# Synergistic Seedling Responses of Distinct Oat Cultivars to Compound Saline–Alkali Stress: Phenotypic, Physiological, and Metabolomic Mechanisms

**DOI:** 10.3390/biology15151275

**Published:** 2026-08-03

**Authors:** Hongna Dou, Xiaoli Wei, Hao Sun, Tingyan Wang, Jing Liu, Wei Wang

**Affiliations:** 1 College of Animal Husbandry and Veterinary Science, Qinghai University, Xining 810016, China; 16697104817@163.com (H.D.); 18894499178@163.com (X.W.); 15373528812@163.com (H.S.); dahailiangci@163.com (T.W.); 2 College of Ecological Environment and Resources, Qinghai Minzu University, Xining 810007, China; jiajing333@163.com; 3 Key Laboratory of Northwest Cultivated Land Conservation and Marginal Land Improvement Enterprises, Ministry of Agriculture and Rural Affairs, Delingha 817000, China

**Keywords:** oat, seedling stage, compound saline–alkali stress, phenotype, physiology, metabolomics

## Abstract

Soil salinization and alkalization severely limit agricultural sustainability in alpine regions. The integrated responses of oats to complex saline–alkali stress, covering phenotypic, physiological, and metabolomic alterations, remain poorly elucidated. This study aimed to clarify the synergistic adaptive mechanisms of oats with differential saline–alkali tolerance. Three oat varieties with distinct tolerance levels, including the tolerant cultivar Meida, the moderately tolerant cultivar Qingtian No. 2, and the sensitive cultivar Qinghai Sweet Oat, were treated with a 150 mmol·L^−1^ mixed saline–alkali solution (Na_2_SO_4_:NaCl:NaHCO_3_ = 2:1:1, pH 8.65) to simulate the natural alpine soil environment of Qinghai Province. Compound saline–alkali stress markedly inhibited oat growth and aggravated cellular oxidative damage. The tolerant cultivar effectively alleviated stress injury by improving antioxidant enzyme activities and accumulating osmoprotectants. A total of 396 core differential metabolites were screened in this study, and flavonoid biosynthesis was identified as a conserved core pathway for oat stress resistance. Tolerant oat varieties simultaneously activated defense responses and energy metabolism to adapt to stress conditions. In contrast, sensitive varieties only depended on basal metabolic adjustments to cope with stress. These findings clarify the differential adaptive strategies of oats under saline–alkali stress. They provide key metabolic marker resources and a solid theoretical basis for the breeding and cultivation of salt–alkali-tolerant oats in alpine saline–alkali land.

## Simple Summary

Soil salinization and alkalinization severely restrict crop growth and agricultural production in cold highland areas. Most previous studies on oats have only focused on single-salt stress, whereas systematic and multi-dimensional investigations of oat responses to compound salt–alkali stress remain limited. In this study, three oat cultivars with distinct salt tolerance levels were selected to investigate their growth performance, physiological responses, and internal metabolic regulation under compound salt–alkali stress that simulates the soil environment of local highland regions. The results indicated that compound salt–alkali stress significantly inhibited oat growth and induced cellular damage. The highly salt-tolerant oat cultivars exhibited superior stress adaptability by enhancing the activities of protective enzymes and accumulating osmoprotective substances. Key metabolic pathways associated with stress tolerance were identified, among which flavonoid biosynthesis played a critical role in stress resistance. Specifically, tolerant oat cultivars activated both defensive response and energy metabolism pathways to cope with stress, while sensitive cultivars only displayed basic passive stress responses. These findings provide valuable insights for the screening and breeding of salt–alkali-tolerant oat cultivars, and support the sustainable development of agriculture in cold highland regions.

## 1. Introduction

Soil salinization has become a primary abiotic stress limiting sustainable agricultural development worldwide. Under the compound impacts of climate change, unsustainable irrigation, and intensive land use, the extent and ecological damage of soil salinization continue to expand, seriously threatening global food security and agricultural sustainability. Recent global saline soil surveys cover agricultural lands in 118 countries, representing 85% of the global land area. Over 424 million hectares of topsoil (0–30 cm) and 833 million hectares of subsoil (30–100 cm) are affected by salinity. Saline soils are mainly distributed in arid deserts and grasslands [1]. Approximately 20% of the world’s arable land and 33% of irrigated farmland are affected by varying degrees of salt stress, leading to agricultural economic losses of up to $27 billion annually due to salinization. It is projected that by 2050, saline-affected land will exceed 50% of the global arable land [2].

In China, the total area of saline–alkali land reaches 1.5 billion mu, of which about 500 million mu has agricultural potential. These lands are mainly distributed in four regions: soda-saline areas in Northeast China, inland saline areas in Northwest China, coastal saline areas, and the Yellow River irrigation region [3,4]. Soil salinization can reduce crop yields by up to 58% [5]. Even low salt concentrations significantly impair crop growth and productivity, while severe salinization may lead to total crop failure, exacerbating the conflict between limited arable land and food security. Salinity and alkalinity stresses damage plants through a tripartite mechanism involving osmotic imbalance, ionic toxicity, and oxidative stress, disrupting cellular water metabolism, ionic homeostasis, and photosynthetic efficiency, ultimately inhibiting plant growth and development [6,7,8]. Therefore, exploring salt-tolerant germplasm and elucidating salt tolerance mechanisms have become core strategic priorities for the comprehensive utilization of saline–alkali land and food security assurance.

Oat is a versatile gramineous crop that integrates ecological remediation, economic value, and nutritional supply. It exhibits superior stress tolerance to cold, drought, and saline–alkali conditions [9]. Accordingly, oat has been widely acknowledged as a promising crop for the remediation of saline–alkali land [8]. Oat can well adapt to ecologically fragile saline–alkali habitats and improve soil physicochemical properties. It regulates soil ion accumulation, reduces surface water evaporation, and inhibits surface salt aggregation, thereby serving as a pioneer crop for the ecological restoration of saline–alkali soils [10]. Furthermore, oat is rich in *β*-glucans, dietary fibers, essential amino acids, and phenolic compounds. It possesses lipid-regulating and cholesterol-lowering functions, conferring broad market application potential [11]. As a polyploid crop with a complex genome, oat harbors abundant genetic resources for stress resistance. Oat copes with compound saline–alkali stress via synergistic strategies, including osmotic adjustment, antioxidant defense, ion homeostasis, and metabolic reprogramming. These characteristics make oat an ideal model crop for exploring plant adaptive mechanisms to compound abiotic stress and breeding saline–alkali-tolerant crop cultivars [12,13]. In addition, oat seedlings present a distinct concentration-dependent response to saline–alkali stress. Low-concentration compound stress only induces mild growth inhibition without disrupting physiological metabolism. A stress concentration of 150 mmol·L^−1^ substantially restricts seedling growth but does not cause plant death. When stress exceeds this critical threshold, seedling growth stagnates and plant mortality gradually occurs [14,15].

Nevertheless, current investigations into oat saline–alkali tolerance have prominent limitations. Most available studies exclusively adopt single NaCl stress treatments, which fail to mimic the native compound saline–alkali soil environment in alpine areas of Qinghai Province [16,17]. Furthermore, relevant works mostly rely on one-dimensional analyses of physiological indices, and few reports integrate phenotypic, physiological, and metabolomic datasets to realize comprehensive multi-dimensional characterization. The genotypic metabolic divergence underlying oats with divergent saline–alkali tolerance also remains insufficiently elucidated, and this knowledge gap hinders optimized cultivation and molecular breeding of salt–alkali-resistant oat cultivars for alpine saline–alkali land. Saline–alkali farmlands across the Qinghai–Tibet Plateau feature naturally coexisting salt and alkali components, while isolated single-salt or single-alkali stress rarely emerges under field conditions. Considering the inherent soil properties of local saline–alkali farmlands in Qinghai, this study targeted the synergistic impacts of compound saline–alkali stress and excluded independent single-salt and single-alkali control groups from the experimental design. In addition, plant stress responses display distinct temporal disparities: endogenous physiological and metabolic alterations arise substantially earlier than macroscopic phenotypic injuries [18]. This experiment applied short-term compound saline–alkali stress. No visible detrimental phenotypes, such as leaf chlorosis and seedling wilting, were detected in oat seedlings, yet latent physiological impairment and metabolic disturbance had already accumulated inside plant tissues. For this reason, the unified term “stress” is used throughout this manuscript to denote the applied treatment condition.

To unify core conceptual definitions in this manuscript, this study strictly differentiates additive effects and synergistic effects induced by compound abiotic stress. A synergistic effect describes a scenario where the total injury from co-occurring multiple stresses is markedly greater than the linear sum of damage triggered by each individual stress, which fundamentally differs from the purely numerical additive effect [19]. Salt and alkali factors interact closely rather than acting independently within inland saline–alkali farmlands across Northwest China. Crop damage under coexisting salt and alkali conditions cannot be simply calculated via summing the impacts of separate single stresses, thereby generating obvious synergistic interactions. This study systematically analyzed oat synergistic responses to compound saline–alkali stress from three layers: growth phenotype, physiological homeostasis, and metabolic regulation. Quantitative discrimination between additive and synergistic damage effects was further conducted to reveal the unique metabolic reprogramming mechanisms of oats exposed to concurrent salt and alkali stress.

Two core scientific questions remain unresolved regarding how oats respond to naturally occurring compound saline–alkali stress under field conditions: First, what shared response patterns and genotype-specific differences in phenotypic traits, physiological homeostasis, and metabolic profiles exist across oat cultivars with contrasting saline–alkali tolerance? And second, which key differential metabolites and metabolic pathways shape genotypic variation in saline–alkali tolerance, and how do these central metabolites coordinately regulate seedling adaptation to compound stress? Accordingly, this study put forward the following research hypothesis: compound saline–alkali stress suppresses oat seedling growth by inducing oxidative injury, osmotic imbalance, and metabolic reprogramming. Oat cultivars with varying saline–alkali tolerance exhibit distinct metabolic regulatory patterns under stress; tolerant cultivars strengthen stress resilience by activating antioxidant systems, optimizing osmotic adjustment, and synchronizing defensive and energy metabolic pathways, while sensitive cultivars cope with stress primarily via passive suppression of basal metabolism. This work systematically investigated the synergistic seedling responses of oats with divergent tolerance at phenotypic, physiological, and metabolomic levels, and identified core differential metabolites and critical regulatory pathways. The findings offer a theoretical foundation and candidate metabolic markers for screening salt–alkali-tolerant oat germplasm, advancing molecular breeding, and guiding field cultivation management in alpine saline–alkali zones.

## 2. Materials and Methods

### 2.1. Experimental Materials

Based on prior germination-stage salt tolerance screening performed by our research team, three oat cultivars with distinct saline–alkali tolerances were chosen: highly tolerant Meida, moderately tolerant Qingtian No. 2, and salt-sensitive Qinghai Sweet Oat [20]. All experimental plant materials were supplied by Qinghai Kairui Ecological Technology Co., Ltd. (Haidong, China). Experiments were carried out in an RXZ-1000 intelligent artificial climate chamber manufactured by Ningbo Jiangnan Instrument Factory (Nanjing, China), located within the firm’s facility in Ping’an District, Haidong City, Qinghai Province, during June 2025. This equipment allows for precise regulation of the photoperiod, temperature, relative humidity, and photosynthetic photon flux density, with continuously adjustable white light in the range of −600 μmol·m^−2^·s^−1^. Cultivation parameters were set to a 16 h light/8 h dark photoperiod [21], a photosynthetic photon flux density of 400 μmol·m^−2^·s^−1^, a temperature of 25 °C, and a relative humidity of 60% ± 5% [22], and detailed background information for each tested cultivar is summarized in Table 1.

### 2.2. Experimental Design

#### 2.2.1. Preparation of Saline–Alkali Solutions

Mixed saline–alkali solutions were prepared to replicate the ionic characteristics of highland saline–alkali farmland soils collected in Qinghai Province, with Na_2_SO_4_ (Sinopharm Chemical Reagent Co., Ltd., Shanghai, China) serving as the dominant salt source and NaCl and NaHCO_3_ (Sinopharm Chemical Reagent Co., Ltd., Shanghai, China) as secondary constituents at a molar ratio of Na_2_SO_4_:NaCl:NaHCO_3_ = 2:1:1 [23]. Preliminary tests revealed that compound saline–alkali stress at 50–100 mmol·L^−1^ generated mild growth inhibition in oats, while exposure to 150 mmol·L^−1^ significantly restricted seedling development without triggering plant death, and 200 mmol·L^−1^ induced severe leaf wilting [20]. For this reason, a concentration of 150 mmol·L^−1^ was chosen as the treatment level for the present experiment.

Analytical-grade inorganic salts (Sinopharm Chemical Reagent Co., Ltd., Shanghai, China) were precisely weighed, dissolved in distilled water, and brought to a constant final volume of 100 mL. The mixed solution was continuously agitated for 30 min with an 85-2 magnetic stirrer (Xinrui Instrument Factory, Changzhou, China) to ensure full solute dissolution. After equilibration at room temperature, the steady pH value of the working solution was measured at 8.65 with a pre-calibrated pH meter (Sinopharm Chemical Reagent Co., Ltd., Shanghai, China). Distilled water served as the control group (CK). All stress treatments adopted identical salt blending proportions to maintain uniform pH values and equivalent stress intensities with the experimental groups.

#### 2.2.2. Screening of Cultivation Substrates and Their Physicochemical Properties

A composite growth substrate mixed with field topsoil, humus, and vermiculite at a volumetric ratio of 2:1:1 was prepared to mimic natural field rhizosphere environments. The field soil was sampled from representative high-altitude saline–alkali farmlands across Qinghai Province. Before pot filling, the substrate was autoclaved at 121 °C for 30 min and subsequently cooled to room temperature. Its primary physicochemical properties were characterized as follows: bulk density 1.22 g·cm^−3^, total porosity 54.2%, pH 7.85, electrical conductivity (EC) 0.22 mS·cm^−1^, and organic matter content 25.8 g·kg^−1^.

Hoagland nutrient solution was omitted in this experiment due to its static nutrient ratios, low ion-buffering performance and fast ion-leaching rate; these traits differ markedly from the intricate colloidal structures, salt-buffering properties, and heterogeneous nutrient profiles inherent to natural saline–alkali soils of Qinghai Province [22,23]. Hydroponic cultivation using nutrient solutions easily induces root hypoxia and abnormal root morphology, which obscures genuine physiological and metabolic responses triggered by compound saline–alkali stress. By comparison, the compound soil substrate maintains the native buffering capacity of field topsoils, supporting gradual ion release alongside balanced aeration and water retention. Its intrinsic organic matter and mineral nutrients eliminate confounding secondary stress artifacts common to hydroponic setups, thus enhancing the authenticity and field representativeness of all measured experimental results [24].

#### 2.2.3. Seedling Stage Experiment

A two-factor experimental design was implemented, consisting of three oat cultivars and two treatment levels (six groups in total); each group included three biological replicates and three technical replicates. Uniform, plump oat seeds were surface-sterilized in 5% sodium hypochlorite (Sinopharm Chemical Reagent Co., Ltd., Shanghai, China) for 10 min, then thoroughly rinsed with sterile distilled water and air-dried. Each pot (inner diameter 20 cm, height 22 cm) was filled with roughly 3 kg substrate, seeded with 50 grains, and covered with a 1 cm soil overlay. At the two-leaf–one-heart seedling stage, excess seedlings were removed to leave 30 evenly developed plants per pot [21]. Irrigation was performed at 09:00 every second day, with each pot receiving 100 mL of either the mixed saline–alkali solution (T1) or distilled water (CK). All leachate draining from the base of each pot was immediately poured back into the corresponding pot to avoid ion depletion. The continuous stress treatment lasted for 14 days. All three cultivars reached the three-leaf–one-heart stage at 14 days after sowing under control conditions.

### 2.3. Measurement Indicators and Methods

#### 2.3.1. Measurement of Phenotypic Indicators

Fourteen days after stress applications, ten evenly developed seedlings were randomly sampled from each pot. All phenotypic traits were quantified across three biological replicates, and mean values were adopted for subsequent statistical analyses. Phenotypic indices were determined via the following procedures. The plant height was measured from the junction of the root and stem as the starting point, and a measuring tape (accuracy 0.1 cm) was used to measure vertically to the tip of the highest leaf of the plant. For the leaf length and width, two fully expanded functional leaves from the oat seedlings at the seedling stage were selected. Leaf length was measured along the naturally oriented leaf from the base of the leaf sheath to the leaf apex. Leaf width was determined at the broadest leaf region after gentle natural flattening without squeezing, with a measurement precision of 0.1 cm.

Aboveground and belowground fresh weights were determined as follows. Ten evenly developed oat plants were harvested, thoroughly rinsed with distilled water, and blotted dry with filter paper to remove surface moisture. Each plant was severed at the root–shoot junction, with stems and leaves defined as the aboveground fraction and the root system as the belowground fraction. Both fractions were weighed individually using an electronic balance (Model MS105DU, Mettler-Toledo Instruments (Shanghai) Co., Ltd., Shanghai, China) with a precision of 0.01 g. Immediately after weighing, all seedling samples were frozen and stored in liquid nitrogen for subsequent physiological and metabolomic analyses.

Synchronous whole-plant phenotypic imaging was carried out using the following procedure. All seedling pots were moved to an imaging platform to guarantee an identical background, shooting height, and camera angle during data collection. Seedlings were raised in the RXZ-1000 artificial climate chamber (Mettler-Toledo Instruments (Shanghai) Co., Ltd., Shanghai, China) under uniform overhead illumination—a condition that kept them growing vertically toward the light source. Manual moving and repositioning of pots disrupted the seedlings’ original upright morphology and introduced random stem tilting in captured images. This uneven inclination did not arise from overwatering, waterlogging-triggered root rot, or seedling transplanting from Petri dishes.

#### 2.3.2. Determination of Physiological Indicators

Physiological and biochemical index quantification procedures were as follows: superoxide dismutase (SOD) activity was assayed via the nitroblue tetrazolium (NBT) photoreduction method [25]; peroxidase (POD) activity was quantified with the guaiacol colorimetric approach [26]; malondialdehyde (MDA) concentration was determined by the thiobarbituric acid colorimetric assay [24]; proline (Pro) content was measured using the ninhydrin colorimetric protocol [27]; soluble sugar levels were quantified through the anthrone colorimetric method [28]; soluble protein concentrations were detected via the Coomassie brilliant blue staining assay [29]; hydrogen peroxide (H_2_O_2_) accumulation was assessed using the titanium salt colorimetric technique [30]; reactive oxygen species (ROS) abundance was analyzed by fluorescence staining [31]; total chlorophyll was extracted with 95% ethanol under dark conditions and quantified by UV–visible spectrophotometry [32]; gibberellin and abscisic acid concentrations were measured using enzyme-linked immunosorbent assay (ELISA) kits (Sinopharm Chemical Reagent Co., Ltd., Shanghai, China) [33]; and root activity was evaluated with the triphenyl tetrazolium chloride (TTC) reduction colorimetric method [34]. All physiological and biochemical measurements were conducted with three technical replicates, and mean values were adopted for subsequent statistical analyses.

### 2.4. Metabolomic Analysis of Oat Seedling Stage: Sample Collection and Extraction Process

#### 2.4.1. Sample Collection and Preservation

Following 14 days of seedling-stage compound saline–alkali stress, six evenly developed seedlings were randomly harvested from each biological replicate (*n* = 3 biological replicates per treatment). Roughly 1 g composite tissue was sampled from the secondary fully functional leaves and primary roots; tissues were snap-frozen in liquid nitrogen for 15 min to terminate cellular metabolic activity and suppress metabolite degradation. After snap-freezing, specimens were transferred to a Haier DW-86L388 (Sinopharm Chemical Reagent Co., Ltd., Shanghai, China) ultra-low-temperature freezer and preserved at −80 °C for subsequent metabolomic profiling.

#### 2.4.2. Sample Preparation

Exactly 30 mg of homogenized sample powder was weighed on a Mettler-Toledo MS105DU (Shanghai Anpu Experimental Technology Co., Ltd., Shanghai, China)electronic balance and transferred into a 2 mL centrifuge tube. A total of 1500 μL of 70% aqueous methanol pre-cooled to −20 °C was added as the extraction solvent, containing the internal standard L-2-chlorophenylalanine (Sigma-Aldrich Co., LLC., St. Louis, MO, USA) at 250 μg·mL^−1^. For samples with an actual mass lower than 30 mg, the solvent volume was proportionally adjusted based on the fixed solvent-to-sample ratio of 1500 μL per 30 mg tissue.

After thorough homogenization of tissue powder in extraction solvent, samples were vortexed for 30 s. The vortex step was repeated six times at 30 min intervals to achieve exhaustive metabolite extraction. The mixture was then centrifuged at 4 °C and 12,000× *g* for 3 min. Exactly 1000 μL supernatant was gently aspirated and passed through a 0.22 μm organic microporous filter membrane (Shanghai Anpu Experimental Technology Co., Ltd., Shanghai, China) to eliminate particulate impurities. The purified filtrate was transferred to a liquid chromatography vial for subsequent ultra-high-performance liquid chromatography–tandem mass spectrometry (UPLC-MS/MS) analysis.

#### 2.4.3. UPLC-MS/MS Analysis Conditions

Chromatographic separation was carried out on a Waters ACQUITY UPLC HSS T3 column (2.1 mm × 100 mm, 1.8 μm, Shanghai Anpu Experimental Technology Co., Ltd., Shanghai, China). The column oven temperature was held at 35 °C, with a mobile phase flow rate of 0.3 mL·min^−1^ and an injection volume of 2 μL. The mobile phase A consisted of ultrapure water with 0.1% formic acid, while mobile phase B was acetonitrile supplemented with 0.1% formic acid. All LC-MS-grade reagents were purchased from Merck (Merck KGaA, Darmstadt, Germany). The linear gradient elution program was configured as follows: 0–1 min, isocratic hold at 5% solvent B; 1–8 min, linear ramp of solvent B from 5% to 95%; 8–10 min, constant elution at 95% solvent B; 10.0–10.1 min, rapid reversion of solvent B from 95% back to 5%; and 10.1–12 min, isocratic hold at 5% solvent B for column re-equilibration.

Mass spectrometric parameters were configured as follows: electrospray ionization (ESI) source temperature, 500 °C; ion spray voltage (IS), 5500 V for positive ionization mode and −4500 V for negative ionization mode; ion source gas 1 (GS1), ion source gas 2 (GS2), and curtain gas (CUR) at 345 kPa, 414 kPa, and 172 kPa, respectively; and collision-induced dissociation intensity was set to high. Triple quadrupole (QQQ) acquisition was run in multiple reaction monitoring (MRM) mode, with collision gas (nitrogen) pressure adjusted to medium. The declustering potential (DP) and collision energy (CE) were individually optimized to obtain characteristic DP and CE values for each MRM ion transition. Distinct groups of MRM ion transitions were monitored in scheduled windows according to the retention time of eluting metabolites.

### 2.5. Data Processing

Raw experimental datasets were compiled in Microsoft Excel 2019. One-way analysis of variance (ANOVA) and Student’s *t*-tests were implemented via Origin 2024 to detect significant differences across treatment groups. Principal component analysis (PCA) and hierarchical cluster analysis (HCA) were performed in R (v4.3.0). Differential metabolites were screened based on two thresholds: variable importance in projection (VIP) > 1 derived from the OPLS-DA model, and *p* < 0.05 from *t*-tests. Phenotypic and physiological data were subjected to two-way ANOVA, with Duncan’s multiple range test applied for post hoc significance separation at *p* < 0.05. All quantitative results are expressed as mean ± standard error (SE).

#### 2.5.1. Phenotypic and Physiological Datasets

Two-way analysis of variance (ANOVA) was used to assess the main effects of oat cultivars, compound saline–alkali treatments, and their interactions on all recorded phenotypic and physiological traits. One-way ANOVA followed by Duncan’s multiple range test was carried out for post hoc pairwise comparisons between the six experimental groups. Independent two-sample Student’s *t*-tests were applied solely for contrasts between the control (CK) and compound saline–alkali stress (T1) within each oat cultivar. Statistical significance was defined at *p* < 0.05 using unadjusted raw *p*-values; a false discovery rate (FDR) correction was applied to all datasets in this study.

#### 2.5.2. Metabolomic Datasets

Multivariate statistical models, namely principal component analysis (PCA) and orthogonal partial least squares discriminant analysis (OPLS-DA), were constructed to profile global metabolic shifts among all samples. FDR-based multiple-testing corrections were applied to *t*-test results to filter false positives generated by high-throughput parallel metabolite testing. Metabolites with significant differential abundance were identified using a compound two-tier screening cutoff: variable importance in projection (VIP) > 1 from the OPLS-DA model, and adjusted FDR < 0.05. Uncorrected raw *p*-values served only as supplementary references and were not adopted as independent criteria for screening differential metabolites.

## 3. Results and Analysis

### 3.1. Effects of Compound Salt–Alkali Stress on Phenotypic Traits of Different Oat Varieties

**Figure 1:**
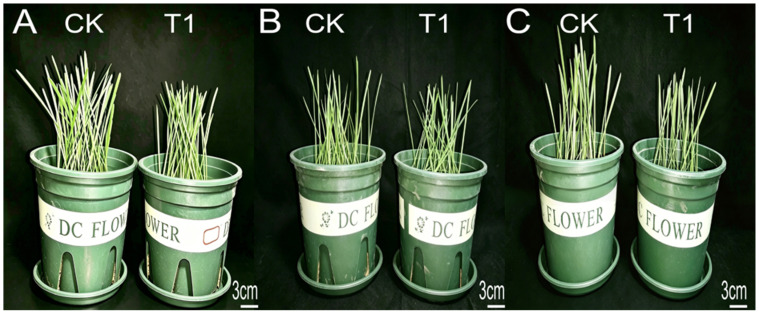
Effects of compound saline–alkali stress on aboveground morphology of seedlings from different oat varieties: (**A**) V4 (Qingtian No. 2); (**B**) V5 (Qinghai Sweet Oat); and (**C**) V15 (Meida). CK indicates the control group irrigated with distilled water, and T1 represents the 150 mmol·L^−1^ compound saline–alkali treatment group with a molar ratio of Na_2_SO_4_:NaCl:NaHCO_3_ = 2:1:1.

**Figure 2:**
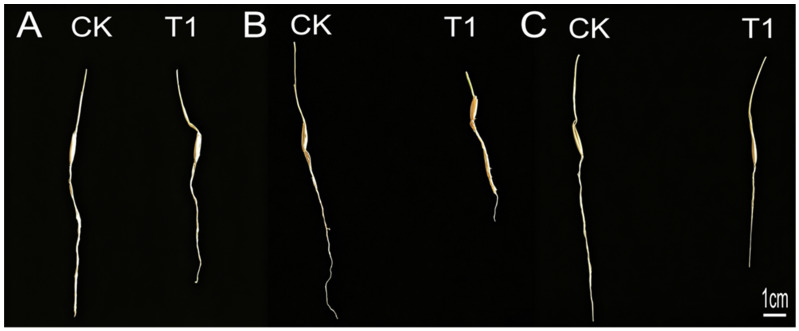
Effects of compound saline–alkali stress on root systems of seedlings from different oat varieties: (**A**) V4 (Qingtian No. 2); (**B**) V5 (Qinghai Sweet Oat); and (**C**) V15 (Meida). CK indicates the control group irrigated with distilled water, and T1 represents the 150 mmol·L^−1^ compound saline–alkali treatment group with a molar ratio of Na_2_SO_4_:NaCl:NaHCO_3_ = 2:1:1.

Three oat cultivars (V4, V5, and V15) were subjected to compound saline–alkali stress. Their morphological phenotypes were recorded and quantified (Figure 1 and Figure 2). Table 2 shows that the stress significantly suppressed seedling growth across all three genotypes, and the magnitude of growth inhibition matched their inherent saline–alkali tolerance. The salt-sensitive genotype V5 displayed the largest decline in biomass; the highly tolerant cultivar V15 retained relatively robust growth; and the moderately tolerant V4 showed intermediate stress-induced damage (as shown in Figure 1 and Figure 2). Compound saline–alkali stress significantly reduced the plant height, root length, and leaf length; the relative reduction rates of these morphological indicators ranged from 17.37% to 62.37%. V4 exhibited the largest decline in leaf length, while V5 had the shortest root systems under stress. In contrast, V15 retained the tallest plants and longest leaves after saline–alkali exposure. Aboveground and root fresh weights declined significantly across all cultivars, with V5 exhibiting the sharpest loss in fresh biomass. In contrast, the leaf width remained unaffected by the compound saline–alkali treatment (*p* > 0.05); only trivial, non-significant increases were observed in V4 and V5. Overall, compound saline–alkali stress strongly restricts vegetative growth of oat seedlings. The tolerant cultivar V15 incurred minimal losses in plant height and biomass, while the sensitive genotype V5 underwent the most pronounced growth retardation. These inter-cultivar phenotypic differences directly reflect genotypic variation in saline–alkali resistance among oats.

### 3.2. Effects of Compound Saline–Alkali Stress on the Physiology of Seedlings of Different Oat Varieties

**Figure 3:**
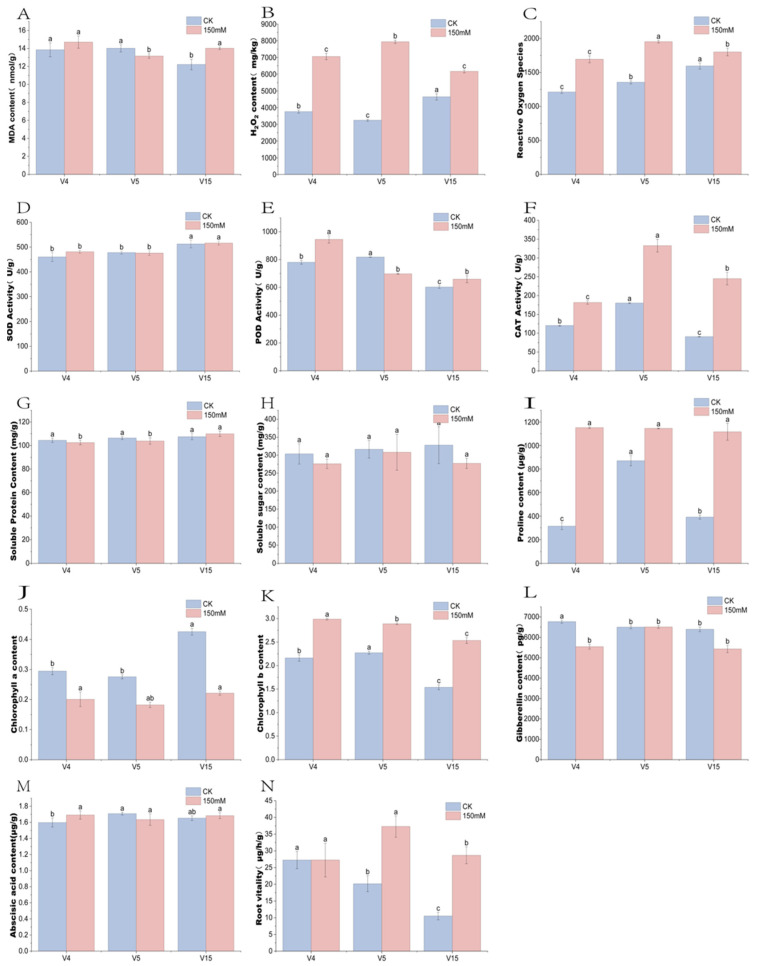
Changes in physiological and biochemical indicators of three oat varieties under controls and 150 mmol·L^−1^ compound saline–alkali stress. (**A**) Malondialdehyde (MDA) content; (**B**) hydrogen peroxide (H_2_O_2_) content; (**C**) reactive oxygen species (ROS) levels; (**D**) superoxide dismutase (SOD) activity; (**E**) catalase (CAT) activity; (**F**) peroxidase (POD) activity; (**G**) soluble sugar content; (**H**) soluble protein content; (**I**) proline content; (**J**) chlorophyll a content; (**K**) chlorophyll b content; (**L**) gibberellin (GA) content; (**M**) abscisic acid (ABA) content; and (**N**) root activity. CK = distilled water control group; 150 mM = compound saline–alkali stress group; and V4, V5, and V15 stand for Qingtian No. 2, Qinghai Sweet Oat, and Meida, respectively. All data are presented as mean ± standard error (SE). Different lowercase letters above bars denote significant differences between groups at *p* < 0.05 based on one-way ANOVA.

The three oat cultivars exhibited distinct seedling physiological responses under 150 mmol·L^−1^ compound saline–alkali stress. Physiological data (as shown in Figure 3) revealed that stress induced leaf accumulation of MDA (as shown in Figure 3A), H_2_O_2_ (as shown in B)and ROS (as shown in C), which aggravated lipid peroxidation of cell membranes. MDA concentrations rose significantly in V4 and V15 under stress relative to CK (*p* < 0.05), with increases of 6.21% and 14.79%, respectively. The H_2_O_2_ content increased by 87.72% (V4), 144.75% (V5), and 32.77% (V15). V5 accumulated the highest H_2_O_2_ and suffered the most serious oxidative damage. The ROS content of V4, V5, and V15 increased by 39.87%, 44.13%, and 12.89%, respectively. Notably, V15 exhibited significantly lower accumulations of H_2_O_2_ and ROS than the other two cultivars. Overall, the salt-sensitive cultivar V5 suffered the most severe oxidative damage, while the highly tolerant cultivar V15 effectively restrained ROS accumulation under saline–alkali stress.

Antioxidant enzyme activities differed significantly among cultivars (*p* < 0.05). Under compound saline–alkali stress, the activities of CAT (as shown in Figure 3F), POD (as shown in Figure 3E), and SOD (as shown in Figure 3D) in cultivar V4 increased by 51.09%, 21.05%, and 4.61%, respectively, relative to CK, with the most pronounced elevation observed in POD activity. For cultivar V5, CAT activity increased sharply by 84.61%, whereas POD activity decreased by 14.7%. CAT, POD, and SOD activities in V15 increased by 170.31%, 9.39%, and 0.69%, respectively. This result indicated that V15 mainly boosts antioxidant capacity through drastic CAT upregulation. Osmoprotectant accumulation also exhibited distinct genotypic differences among cultivars. In V15, soluble protein and free proline contents were significantly upregulated (*p* < 0.05), with increases of 2.33% and 185.41%, respectively. Cultivar V4 showed the greatest proline accumulation, with a 266.70% increase; however, its soluble sugar content decreased significantly by 8.96% (*p* < 0.05), while its soluble protein content remained unaltered. In cultivar V5, its soluble sugar and soluble protein levels declined significantly by 2.39% and 2.54% (*p* < 0.05), whereas proline content was only slightly elevated under stress.

Chlorophyll and endogenous hormone analyses revealed significant declines in chlorophyll a (as shown in Figure 3J) across all three cultivars under stress (*p* < 0.05), with reduction rates of 31.82% (V4), 33.91% (V5), and 48.04% (V15). In contrast, chlorophyll b (as shown in Figure 3K) levels increased significantly (*p* < 0.05). V15 maintained the smallest drop in the chlorophyll a/b ratio. Hormonal measurements showed that gibberellin (GA) (as shown in Figure 3L) content was significantly reduced in V5 (*p* < 0.05), while abscisic acid (ABA) (as shown in Figure 3M) content was significantly elevated in all three cultivars, with incremental amplitudes of 38.25%, 27.23%, and 64.76%, respectively. The saline–alkali-tolerant cultivar V15 retained a more stable chlorophyll a/b ratio and displayed weaker stress-induced growth inhibition.

### 3.3. Metabolomic Analysis of Different Oat Varieties Under Composite Salt–Alkali Stress

#### 3.3.1. Quality Control and Overall Characteristics of Metabolomic Data

**Figure 4:**
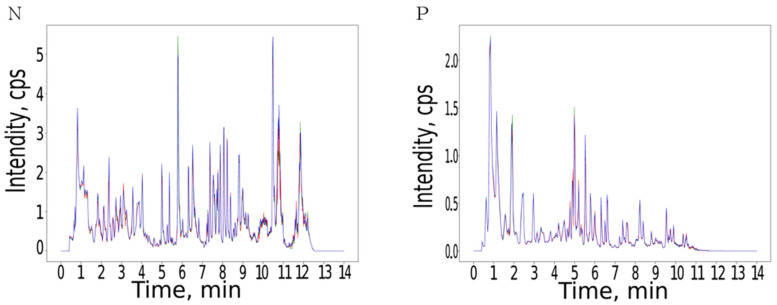
Total ion chromatograms (TICs) of quality control (QC) samples from UPLC-MS/MS metabolomic detection. P represents positive ion mode; N represents negative ion mode.

Total ion chromatograms (TICs) of QC samples fully overlapped, with consistent retention times and peak intensities (as shown in Figure 4). These profiles confirmed stable instrument performance and high repeatability of the detection method. The metabolomic dataset was thus reliable for subsequent screening of differential metabolites.

**Figure 5:**
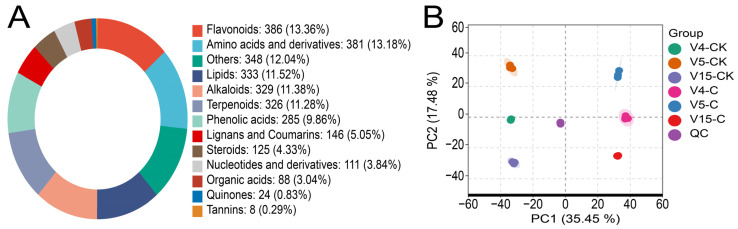
Overview of metabolomic profiles of oat seedling samples under control and compound saline–alkali stress. (**A**) Pie chart illustrating the classification and proportion of all detected metabolites; (**B**) two-dimensional PCA score plot showing sample separation of three oat cultivars under CK and T1 treatments.

Thirteen major categories of primary and secondary metabolites were annotated, including alkaloids, amino acid derivatives, flavonoids, lipids, terpenoids, phenolic acids, lignans, coumarins, organic acids, and nucleotide derivatives. All annotated metabolites cover core metabolic pathways associated with plant stress adaptation. Amino acids and its derivatives (13.18%), flavonoids (13.18%), lipids (11.52%), alkaloids (11.28%), terpenoids (11.28%), and other compounds (12.04%) were the predominant components, constituting the core metabolic network of oat seedlings. Principal component analysis (PCA) revealed that PC1 and PC2 collectively explained 52.93% of the total metabolic variance. In the PCA score plot, samples from different oat cultivars and treatment groups exhibited clear separation, whereas biological replicates within the same group clustered closely (as shown in Figure 5). These results confirmed high experimental reproducibility and robust data reliability, providing a credible foundation for subsequent differential metabolite screening.

**Figure 6:**
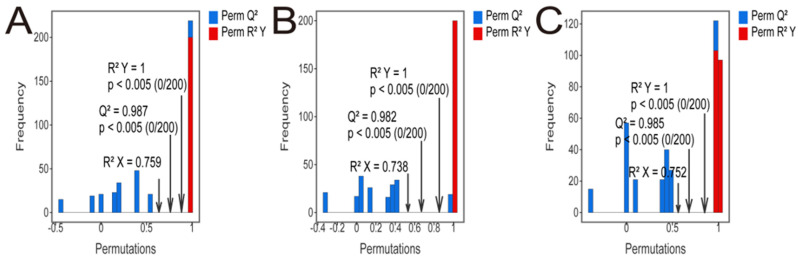
Permutation test results of OPLS−DA models for three oat cultivars. (**A**) V4 (Qingtian No. 2); (**B**) V5 (Qinghai Sweet Oat); and (**C**) V15 (Meida).

Permutation tests for the OPLS−DA models (as shown in Figure 6) confirmed the absence of overfitting across all three oat cultivars. Key model performance metrics are listed as follows: the V4 model yielded R^2^X = 0.759, R^2^Y = 1, and Q^2^ = 0.987; the V5 model yielded R^2^X = 0.738, R^2^Y = 1, and Q^2^ = 0.982; and the V15 model yielded R^2^X = 0.752, R^2^Y = 1, Q^2^ = 0.985. All models attained Q^2^ > 0.98, reflecting strong explanatory and predictive power for sample grouping and validating their use for subsequent differential metabolite screening.

R^2^Y = 1 and Q^2^ > 0.98 were rarely observed in standard plant metabolomic datasets. The model’s strong discrimination power originated from large metabolic differences between control and stressed seedlings. The three biological replicates for each treatment also minimized intra-group metabolic variation. Notably, all permutation regression lines displayed negative Q^2^ intercepts, a critical diagnostic indicator ruling out model overfitting. Overall, permutation results validated that the clear sample separation shown in OPLS−DA score plots stemmed from genuine stress-induced metabolic reprogramming instead of artificial bias caused by model overfitting.

#### 3.3.2. Differential Metabolite Screening and Variety-Specific Characterization

**Figure 7:**
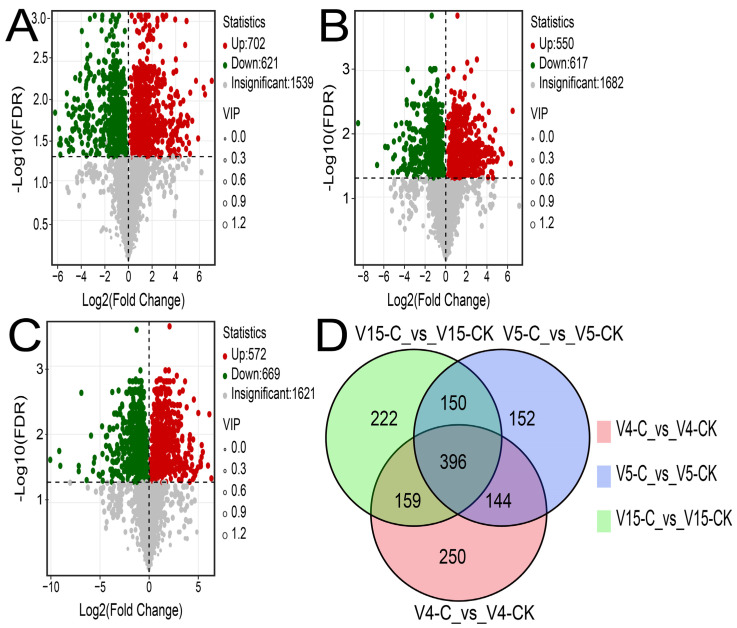
Screening and overlap analyses of significantly differential metabolites (SDMs) in three oat cultivars under compound saline–alkali stress. (**A**–**C**) Volcano plots of SDMs for V4, V5, and V15. Red dots denote upregulated metabolites; blue dots denote downregulated metabolites; and gray dots denote non-differential metabolites (screening thresholds: VIP > 1, FDR < 0.05). (**D**) Venn diagram illustrating shared and unique SDMs across the three cultivar comparison sets.

Differential metabolites were screened based on the dual thresholds VIP > 1 and FDR < 0.05. A total of 1323 significantly altered metabolites were annotated in cultivar V4, including 702 upregulated and 621 downregulated compounds, with upregulated metabolites dominating the profile. For cultivar V5, 1167 differential metabolites were detected: 550 upregulated and 617 downregulated, revealing a greater abundance of repressed metabolites. Cultivar V15 yielded 1241 significantly differentially abundant metabolites, of which 572 were upregulated and 669 were downregulated, corresponding to a larger pool of downregulated metabolites (as shown in Figure 7).

Venn diagram analyses (as shown in Figure 7) visualized the distribution of differential metabolites across three comparison groups: V4-C vs. V4-CK, V5-C vs. V5-CK, and V15-C vs. V15-CK. Each group contained unique differential metabolites: 250 in V4, 152 in V5, and 222 in V15. The number of shared metabolites between pairwise groups were 144 (V4 and V5), 159 (V4 and V15), and 150 (V5 and V15). A total of 396 metabolites were shared by all three cultivars. These shared metabolites accounted for 41.73% of V4’s total differential metabolites, 47.03% of V5’s, and 42.72% of V15’s.

#### 3.3.3. Differential Metabolite KEGG Pathway Enrichment Analysis

**Figure 8:**
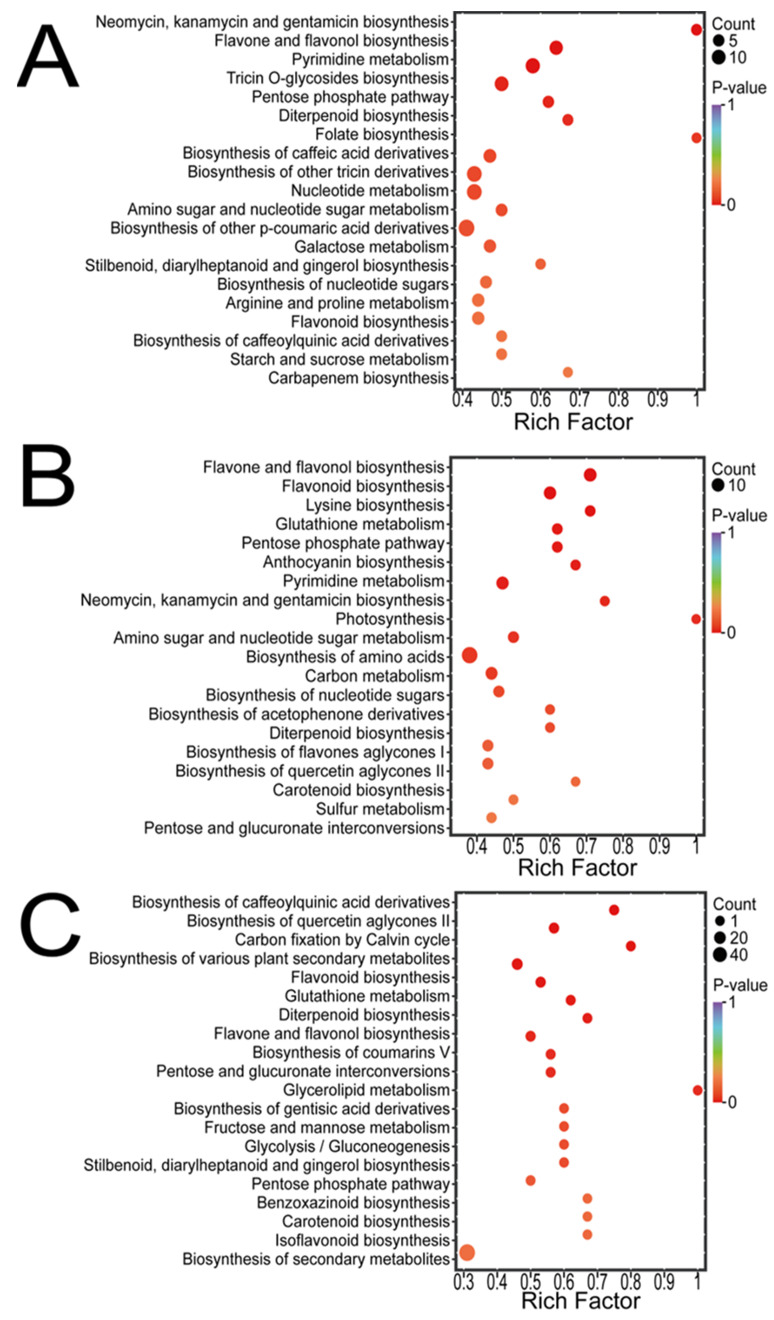
Bubble plots illustrating KEGG pathway enrichment of differential metabolites from three oat cultivars under compound saline−alkali stress. (**A**) V4; (**B**) V5; and (**C**) V15. The x-axis denotes the rich factor; the bubble size corresponds to the count of enriched differential metabolites; the color gradient indicates enrichment significance, with red representing the highest significance and blue the lowest. Enrichment is deemed significant at *p* < 0.05 and highly significant at *p* < 0.01.

KEGG pathway enrichment (as shown in Figure 8) identified five significantly enriched core pathways for differential metabolites (*p* < 0.05): flavonoid biosynthesis, secondary metabolite biosynthesis, pentose phosphate pathway, glutathione metabolism, and terpenoid biosynthesis. The three cultivars showed distinct enrichment patterns. Differential metabolites of V4 were significantly enriched in aminoglycoside antibiotic biosynthesis, triterpene O-glycoside biosynthesis, and arginine and proline metabolism (*p* < 0.01). V5 displayed extreme enrichment exclusively in flavonoid biosynthesis (*p* < 0.01). Metabolites from V15 exhibited highly significant enrichment in biosynthesis of secondary metabolites (*p* < 0.01); multiple stress defensive pathways, including phenylpropanoid metabolism, flavonoid biosynthesis, and glutathione metabolism, also showed prominent enrichment (*p* < 0.01).

## 4. Discussion

### 4.1. Differences in Responses to Compound Saline–Alkali Stress and Single-NaCl Stress

Compound saline–alkali stress triggers concurrent ionic toxicity, osmotic injury, and high-pH alkali stress [35,36]. Its regulatory mechanisms are far more intricate and its plant adaptive responses more specific, relative to single-NaCl stress. The present study revealed bidirectional reprogramming of alkaloid metabolism in oat seedlings exposed to compound saline–alkali conditions, accompanied by marked accumulation of organic acids; such metabolic shifts represent key diagnostic traits differentiating compound saline–alkali stress from single-salt stress. Furthermore, the flavonoid biosynthesis pathway remained highly conserved across oat cultivars with divergent saline–alkali tolerance, consistent with Ma et al. [37], who identified flavonoids as central stress-defensive metabolites in oat metabolomic analyses under abiotic stress. This evidence validates that flavonoids function as pivotal metabolites mitigating oxidative damage induced by compound saline–alkali stress, and can be adopted as core metabolic biomarkers for oat stress responses to mixed saline–alkali environments. Collectively, these findings elucidate fundamental disparities in plant regulatory networks between compound saline–alkali stress and isolated NaCl treatment, overcoming the narrow scope of prior research centered exclusively on single-NaCl salinity.

### 4.2. Physiological Responses Differentiation of Different Salt-Tolerant Oat Varieties

Salt stress triggers massive accumulation of reactive oxygen species (ROS), which further causes membrane lipid peroxidation and cellular injury. Activation of the antioxidant system and buildup of osmoprotectants constitute core adaptive strategies, whereby plants counteract salt-induced injury and sustain physiological homeostasis [38,39]. The present study demonstrated that under compound saline–alkali stress, salt-sensitive cultivar V5 displayed pronounced elevations in malondialdehyde, hydrogen peroxide, and ROS concentrations, accompanied by the most severe membrane lipid peroxidation. Meanwhile, its peroxidase activity declined, and the insufficient accumulation of osmoprotectants, including proline and soluble sugars, ultimately triggered marked growth suppression. In comparison, the highly tolerant cultivar V15 efficiently restrained ROS overaccumulation via striking upregulation of catalase activity and elevated proline concentrations. This genotype mitigated stress-induced damage by boosting antioxidant activity and reinforcing osmotic adjustment, thereby sustaining relatively steady seedling growth. The moderately tolerant cultivar V4 exhibited intermediate physiological phenotypes, with a modest activation of antioxidant enzymes and a moderate accumulation of osmoprotectants, alongside milder growth retardation. Distinct physiological responses of these biomarkers clearly reflect genotypic divergence in the antioxidant potential and osmotic adjustment efficiency among oat cultivars with varying saline–alkali tolerance, consistent with the physiological patterns reported by Gao et al. [8] in oat root systems exposed to mixed saline–alkali stress. Notably, latent physiological and metabolic perturbations that lack obvious visible morphological lesions account for the apparent disconnect between seedling phenotypic photographs and the terminology “saline–alkali stress” adopted herein. Early plant stress responses primarily manifest at physiological and metabolic layers, whereas macroscopic symptoms, such as stunted growth, leaf wilting, and chlorosis, develop far later. Even in the absence of intuitive stress phenotypes in morphological images, severe disruption of ROS homeostasis, osmotic equilibrium, and central metabolic pathways verified that oat seedlings sustained substantial stress damage. Therefore, the consistent use of the term “compound saline–alkali stress” throughout this manuscript remains scientifically rigorous and appropriate; this supplementary clarification resolves potential reader confusion in linking plant morphology to stress nomenclature.

### 4.3. Metabolomic Reprogramming of Oat Under Complex Saline–Alkali Stress: Conserved Pathways and Variety-Specific Regulation

Metabolomic profiling reveals that flavonoids, amino acid derivatives, lipids, and alkaloids constitute the core differential metabolites mediating oat responses to compound saline–alkali stress. The flavonoid biosynthesis pathway was significantly enriched in all three cultivars, serving as a highly conserved stress defensive pathway. Flavonoids exert multiple biological functions, including antioxidant activity, ROS scavenging, and membrane stabilization [39], and their persistent upregulation represents a crucial adaptive strategy for oats to alleviate oxidative damage under compound saline–alkali conditions. In addition, polyols were markedly accumulated under saline–alkali stress and functioned as essential osmoprotectants and antioxidants. Their accumulation patterns were consistent with the previous findings reported by Pamuru et al. [40], who demonstrated that polyols are vital for maintaining plant osmotic homeostasis under stress, further confirming the indispensable role of polyol metabolites in oat saline–alkali tolerance. Distinct metabolic reprogramming was observed among cultivars with different stress tolerances. Previous studies have indicated that the downregulation of carbon and nitrogen metabolism dominates salt stress damage in naked oats under single NaCl stress [41]. In comparison, the present study has newly revealed that the highly tolerant cultivar V15 simultaneously activates flavonoid-dependent secondary defense pathways and energy metabolism pathways, including glycolysis and the Calvin cycle, thereby achieving synergistic stress defense and energy supply. The moderately tolerant cultivar V4 mainly accumulated secondary metabolites, such as flavonoids and phenolic acids, whereas the sensitive cultivar V5 passively adjusted basal metabolic processes, including photosynthesis and carbon metabolism. The present study systematically elucidates this genotypic metabolic differentiation under compound saline–alkali stress for the first time, which complements the previous findings regarding metabolic inhibition induced by single-NaCl stress [42]. These results highlight that the stress type serves as a decisive factor shaping distinct metabolic regulatory patterns in oat seedlings.

### 4.4. Limitations and Future Directions

This study applied only a fixed salt mixture ratio with a stable pH of 8.65 to establish compound saline–alkali stress treatments, without setting gradient groups with varying salt proportions. Therefore, the individual contributions of specific salt ions and solution alkalinity to oat growth inhibition and metabolic reprogramming cannot be quantitatively distinguished. In addition, the experimental design simulated natural compound saline–alkali conditions of high-altitude farmlands and lacked independent single-salt and single-alkali treatment groups. Accordingly, the respective effects of chloride–sulfate salt stress and sodium bicarbonate-induced alkali stress on oat growth and metabolic remodeling could not be separately quantified.

To compensate for these experimental deficiencies, subsequent studies will add independent treatment groups, including single-chloride salt stress, single-sulfate salt stress, and single-sodium-bicarbonate alkali stress. Comparative analyses of phenotypic, physiological, and metabolomic variations among single-salt, single-alkali, and compound stress treatments will help disentangle the independent effects of salt and alkali stress and clarify their synergistic regulatory mechanisms in oat seedlings. Such follow-up investigations will further improve the theoretical system of oat stress adaptation under natural complex saline–alkali environments.

Furthermore, the tolerant-related metabolic biomarkers screened in the present study were obtained merely from three oat cultivars under a single simulated saline–alkali condition, and their universal applicability remains unconfirmed. Future research will expand the oat germplasm collection and conduct multi-site field validation in diverse high-altitude saline–alkali soils, aiming to verify the stability and broad applicability of these core metabolites under variable field habitats.

## 5. Conclusions

This study investigated seedling-stage synergistic responses to compound saline–alkali stress using three oat cultivars with distinct tolerance levels collected from high-altitude regions of Qinghai. Compound saline–alkali stress markedly suppressed oat growth and exacerbated oxidative damage. The highly tolerant cultivar Meida alleviated stress injury through enhanced antioxidant defense and efficient osmotic adjustment, whereas the sensitive cultivar Qinghai Sweet Oat exhibited the most severe physiological damage and the lowest stress adaptability. Metabolomic screening identified flavonoids, amino acid derivatives, lipids, and alkaloids as the core differential metabolites, among which flavonoid biosynthesis served as a conserved stress tolerance pathway. The tolerant cultivars concurrently activated defensive secondary metabolism and energy metabolism, while the sensitive cultivars primarily adopted passive adjustment of basal metabolic processes. Overall, phenotypic, physiological, and metabolic responses formed a synergistic regulatory network underlying oat stress adaptation. This study systematically elucidates the genotype-dependent metabolic reprogramming strategies of oats under high-altitude compound saline–alkali stress and characterizes key stress-responsive metabolites and core regulatory pathways. These findings provide a fundamental theoretical basis for saline–alkali-tolerant germplasm breeding and cultivation optimization for oats in high-altitude saline–alkali land.

## Figures and Tables

**Table 1:** Oat test materials and their identification numbers.

Variety Number	Name	Thousand Grain Weight
V4	Qingtian No. 2	35.6 g
V5	Qinghai Sweet Oat	68.7 g
V15	Meida	31.0 g

**Table 2:** Effects of compound salt–alkali stress on phenotypic indicators of different oat varieties.

Variety Number	Treatments	Plant Height (cm)	Root Length (cm)	Leaf Length (cm)	Leaf Width (cm)	Aboveground Fresh Weight (g)	Belowground Fresh Weight (g)
V4	CK	20.73 ± 1.36 c	6.23 ± 0.15 c	13 ± 1.83 b	0.3 ± 0 b	1.1 ± 0.2 c	0.61 ± 0.11 c
V5	CK	24.23 ± 1.8 b	7.1 ± 0.1 a	12 ± 0.6 c	0.37 ± 0.06 ab	1.37 ± 0.06 b	0.75 ± 0.04 b
V15	CK	29.17 ± 1.72 a	6.87 ± 0.06 b	21.6 ± 1.4 a	0.3 ± 0 b	1.53 ± 0.06 a	0.88 ± 0.04 a
V4	T1	17.8 ± 0.82 f	5.13 ± 0.15 d	5 ± 0.36 f	0.37 ± 0.06 ab	0.67 ± 0.12 e	0.39 ± 0.08 e
V5	T1	14.97 ± 0.71 e	4.73 ± 0.06 e	6.3 ± 0.5 e	0.4 ± 0 a	0.53 ± 0.15 f	0.3 ± 0.09 f
V15	T1	19.23 ± 1.75 d	4.87 ± 0.06 f	10.7 ± 0.46 d	0.33 ± 0.06 ab	0.95 ± 0.05 d	0.55 ± 0.03 d

(Note: Data are presented as mean ± standard error; different lowercase letters in the same column indicate significant differences between treatments (*p* < 0.05).)

## Data Availability

The original contributions presented in this study are included in the article. Further inquiries can be directed to the corresponding author.

## Abbreviations

The following abbreviations are used in this manuscript:

MDA	Malondialdehyde
H_2_O_2_	Hydrogen Peroxide
ROS	Reactive Oxygen Species
CAT	Catalase
POD	Peroxidase
SOD	Superoxide Dismutase
GA	Gibberellin
ABA	Abscisic Acid
